# Topical Piperine for the Management of Burning Mouth Syndrome: A Case Series

**DOI:** 10.3390/jcm15103789

**Published:** 2026-05-14

**Authors:** Elena M. Varoni, Marco Meregalli, Giovanni Lodi, Sara Vitalini, Marcello Iriti, Andrea Sardella

**Affiliations:** 1 Department of Biomedical, Surgical and Dental Sciences, University of Milan, Via Beldiletto 1, 20142 Milan, Italy; marcomere99@libero.it (M.M.); giovanni.lodi@unimi.it (G.L.); sara.vitalini@unimi.it (S.V.); marcello.iriti@unimi.it (M.I.); andrea.sardella@unimi.it (A.S.); 2 Odontostomatological Units, San Paolo Hospital, ASST Santi Paolo and Carlo, Via Beldiletto 1, 20142 Milan, Italy

**Keywords:** burning mouth syndrome, piperine, topical administration

## Abstract

**Background**: Burning Mouth Syndrome (BMS) is a chronic pain characterized by persistent oral burning and itching sensations without identifiable organic causes. In this case series, we explored the role as non-pharmacological therapy effects of topical piperine in reducing BMS-related symptoms. **Methods**: BMS patients performed a 1 min mouthrinse, twice daily for 8 weeks, with 10 mg of piperine in 20 mL of water. At baseline and post-treatment, pain intensity was evaluated using the Numeric Rating Scale (NRS), while oral-health-related quality of life and sleep quality were assessed using the oral health impact profile (OHIP-14) and Epworth Sleepiness Scale (ESS) questionnaires. **Results**: After 8 weeks of treatment, 9 of 14 patients (64%) reported improvement in BMS symptoms. Pain intensity significantly decreased (NRS scores reduced from 5.10 ± 1.81 to 3.21 ± 2.12; *p* = 0.001). OHIP-14 scores also improved (from 17.6 ± 8.9 to 12.3 ± 10.4; *p* = 0.003), as did ESS scores (from 3.86 ± 3.16 to 2.86 ± 2.96; *p* = 0.008). No adverse events or symptom worsening were reported. **Conclusions**: Topical piperine may help relieve symptoms in BMS, but the evidence is preliminary due to the small sample size and lack of a control group. Randomized controlled trials are needed to explore its effectiveness.

## 1. Introduction

Burning mouth syndrome (BMS), which has been identified over time with multiple terms such as stomatodynia, glossodynia, burning tongue, and oral dysesthesia, is a complex idiopathic orofacial pain disorder characterized by persistent and spontaneous oral discomfort lasting longer than three months, in the absence of identifiable local or systemic pathological alterations [1,2]. The pain is often described as a burning sensation, especially at the tip of the tongue, the anterior third of the palate and the lips, although it can affect any intraoral mucosal area. Patients often report, in addition to burning, which remains the most frequent feature, a variety of concomitant intraoral and extraoral symptoms, which can hinder clinical diagnosis and contribute to diagnostic delays. The global prevalence of BMS is approximately 1.7% when considering the general population, with a predilection for the female sex (female to male ratio of approximately 3:1). Estimates increase to approximately 7.7% when considering patient populations referring to secondary care centers at specialist dental clinics, taking into account, however, that the reported prevalence rates vary widely due to the great heterogeneity between studies, in terms of patient types, geographical regions and diagnostic criteria applied [3]. BMS can be considered a multifactorial disorder, whose pathogenesis is currently believed to be predominantly neuropathic, i.e., related to a dysfunction of the peripheral and central trigeminal pathways [1]. Alterations in the transmission of nociceptive signals and in sensitization occur, resulting in increased neuronal excitability that produces the consequent persistent oral burning; the clinical variability of BMS is then influenced by psychological, hormonal and systemic factors [4].

The diagnosis of BMS is by exclusion and includes ruling out local or systemic conditions that may be associated with oral burning and that can be objectified through clinical examination and diagnostic tests. A complete evaluation, in fact, includes a careful anamnesis (including medications taken, lifestyle, psychological state and comorbidities), detailed intraoral objective examination and laboratory tests that can help exclude underlying causes [1]. The characterization of pain (onset, duration, location, exacerbating or relieving factors) and associated extraoral symptoms represents a crucial aspect to correctly manage the patient. BMS often presents with mild morning symptoms that worsen throughout the day and can be relieved by eating [2]. Patients frequently experience additional symptoms, such as xerostomia, taste alterations, and sensory changes.

Chronic neuropathic pain in BMS can be managed with a variety of non-pharmacological approaches, such as cognitive behavioral psychotherapy or acupuncture, and pharmacological agents, including antidepressants, ion channel blockers, tramadol, opioids, capsaicin, and local anesthetics, though no standardized gold-standard therapy exists [5,6,7,8,9,10]. These treatments alleviate pain via multiple mechanisms, such as neurotransmitter modulation, ion channel blockade, anti-inflammatory effects, and nociceptor desensitization [5], with the most commonly used approaches being topical anesthetics, capsaicin, or benzodiazepines [8]. Capsaicin, in particular, has been proposed in the form of gels and rinses, since this compound is a selective agonist of the transient receptor potential vanilloid 1 (TRPV1) receptor, abundantly expressed on peripheral sensory nerve fibers involved in pain transmission [11]. In BMS, TRPV1 has been reported to be overexpressed at the oral mucosa, contributing to increased nociceptive signals and consequent burning sensations [12]. Topically applied capsaicin, in the form of rinses or gels, is able to initially activate TRPV1 channels on nociceptors of C and Aδ fibers, causing an influx of calcium and sodium ions that correlates with transient burning; continuous or repeated stimulation produces the influx of calcium that is associated with the defunctionalization and desensitization of nociceptive nerve endings, temporary blockade of signal transduction, depletion of neuropeptides such as substance P, and reduced membrane excitability [13]. This results in a sustained reduction in pain perception over time.

Piperine, the main alkaloid of *Piper nigrum* and a cinnamamide derivative, is the principal pungent compound in black pepper and exhibits anti-inflammatory, analgesic, antioxidant, and neuroprotective properties [14,15,16]. It is also found in *Piper longum* L. and demonstrates immune-regulating effects, with potential therapeutic applications in conditions such as sciatica [17], cancer-associated pain [15,18], and rheumatoid arthritis [16]. Pharmacological studies indicate that piperine can inhibit inflammation and nerve-related bone destruction, although the precise mechanisms remain incompletely understood [13,14,15,16]. Similar to capsaicin, piperine acts as a TRPV1 receptor agonist, inducing receptor desensitization, which may underlie its analgesic effects and suggest a role in treating burning mouth syndrome [19]. Preclinical models of neuropathic pain further support its antinociceptive and anti-inflammatory activity: in a paclitaxel-induced neuropathy mouse model, piperine alleviated thermal hyperalgesia and reduced inflammatory (IL-6, TNF-α) and oxidative stress markers [18]. It has also shown efficacy in standard pain assays, such as tail-flick and formalin tests, and may enhance analgesic effects when combined with other bioactive compounds without causing central nervous system side-effects [20]. These findings highlight piperine as a promising candidate for neuropathic pain management, though controlled clinical studies are needed to confirm efficacy and determine optimal dosing.

Our research question aimed at addressing whether, in patients with Burning Mouth Syndrome (BMS), a course of topical piperine administered as a mouthrinse could reduce pain intensity through TRPV1 receptors’ desensitization. This rationale is grounded in the mechanism of capsaicin, a TRPV1 agonist that induces receptor activation followed by desensitization and a consequent reduction in neuropathic pain. Piperine, as a structurally and functionally similar compound, is also capable of activating TRPV1 channels, suggesting that repeated topical exposure may similarly promote receptor desensitization and reduce the characteristic burning symptoms of BMS. The aim of this case series, in particular, was to explore preliminary the effect of 8 weeks topical piperine application, as mouthrinse, on pain relief in patients with BMS, based on its potential to induce TRPV1 receptor desensitization and reduce burning symptoms.

## 2. Materials and Methods

This study was designed as a prospective case series, aimed at exploring the potential role of topical piperine in the management of BMS.

*Population*—Adult patients (over 18 years old) referred to the dental clinic of the ASST Santi Carlo e Paolo Hospital in Milan with a confirmed diagnosis of BMS, according to International Classification of Orofacial Pain (ICOP) Research Diagnostic Criteria (ICOP-1) [21], were enrolled from October 2023 to June 2024. Inclusion criteria were: daily recurring oral pain lasting more than 2 h per day for over 3 months; pain characterized by a burning quality and perceived as superficial within the oral mucosa; clinically normal oral mucosa; exclusion of local or systemic causes; and symptoms not better explained by another ICOP or ICHD-3 diagnosis [21]. Exclusion criteria included: minors; patients with suspected or confirmed hypersensitivity to piperine; patients suffering from ulcers, gastritis, or other gastrointestinal disorders (including Crohn’s disease, diverticulitis, gastritis, or hemorrhoids); patients receiving phenytoin (used in the treatment of epilepsy); patients receiving theophylline (a bronchodilator); patients receiving beta-blockers or ACE inhibitors; patients receiving warfarin or heparin; patients receiving hypoglycemic agents (e.g., metformin, glibenclamide), due to the risk of enhanced hypoglycemic effects; hyposalivation (assessed via unstimulated whole saliva (UWS) flow measurement); and the presence of diabetes, Sjögren Syndrome and thyroid disorders, as well as micronutrient deficiencies (in accordance with exclusion diagnostic criteria for BMS).

*Intervention*—After obtaining informed consent, patients were asked to rinse their mouth for 1 min by dissolving 1 tablet, commercially available, of Piper nigrum 10 mg (95% pure piperine) in 20 mL of water, twice a day (morning and afternoon), for 1 min for 8 weeks. The timespan was sufficient to evaluate the effects of a topical treatment, consistently with previous studies [21]. Participants received standard instructions regarding oral hygiene practices and dietary habits during the study period; in particular, they were asked to avoid oral mouthwashes and the consumption of spicy and acid foods.

*Data collection at baseline—*At baseline, each patient was asked to fill out a questionnaire about socio-demographic data (i.e., sex, age), co-morbidities, drug therapies and lifestyles, including smoking habits and excessive alcohol consumption (exceeding 150 mL of wine or beer or 15 mL of spirits per day). A further questionnaire was used to assess the characteristics of pain and further BMS-associated symptoms: presence and type of pain (i.e., burning, stinging), presence of xerostomia, taste alterations (especially bitter/metallic taste), and dysphagia. The Oral Health Impact Profile (OHIP-14) questionnaire [22] was also used to assess the patient’s quality of life, while the Epworth Sleepiness Scale (ESS) questionnaire [23] to measure quality of sleep. The level of pain intensity perceived by the patient was evaluated using the 0–10 Numeric Rating Scale (NRS) with facial visual anchors, where 0 indicates no pain and 10 the worst imaginable pain, a validated and widely used tool for subjective pain evaluation [24,25]. Oral examination was performed and salivary flow was measured using spitting method. Patients were instructed to let the tablet dissolve in the mouth, then spit it out without swallowing; afterward, they did not rinse, drink, or eat for at least 30 min in order to promote the absorption of piperine through the oral mucosa.

*Data collection at follow-up—*At the end of the 8-week treatment, the patient received a follow-up visit, completing the questionnaires on pain characteristics and further BMS-associated symptoms, OHIP-14, ESS and NRS, as described for baseline evaluation. Oral examination was also performed, and adverse effects related to the treatment were recorded.

*Primary outcomes*—The primary outcomes included the changes in pain intensity over time, after treatment, assessed using NRS. In addition, a clinical response was operationally defined as a reduction of ≥2 points on the NRS [26].

*Secondary outcomes—*The secondary outcomes included: improvements in BMS-related concomitant symptoms, i.e., xerostomia, dysgeusia and dysphagia (recorded as “presence/absence”); improvements of scores on quality of life and quality of sleep based on OHIP-14 and ESS questionnaires, respectively; and occurrence of adverse events or side-effects related to topical piperine.

*Statistical analysis—*Descriptive variables were expressed as mean ± standard deviation (SD) for continuous variables with a normal distribution, and as median and 25th and 75th percentile values for variables with a non-normal distribution. Dichotomous variables were reported as numbers and percentages. Normality of the differences between pre- and post-treatment measurements was assessed using the Shapiro–Wilk test. In cases of non-normal distribution, the Wilcoxon signed-rank test was applied, whereas for normally distributed data, a paired samples *t*-test for dependent samples with 95% confidence interval was used (comparing the data obtained from patients before using piperine and the data obtained after the 8-week treatment). All statistical analyses were performed using OriginPro 2026 (Northampton, Massachusetts, USA). Statistical analyses were performed using Excel^®^ Version 2603 (Microsoft 365, Redmond, Washington, USA), and statistical significance was set at *p* ≤ 0.05. All analyses were performed, verified, and interpreted by the authors.

## 3. Results

### 3.1. Socio-Demographic Data

34 patients with BMS were examined, and 17 of them were excluded since they had one or more exclusion criteria. Therefore, 17 patients were finally enrolled in the case series: 15 women (88%) and 2 men (12%). The average age was 71.9 ± 10.8 years, with a median age of 75 years.

Three patients were lost at follow-up, representing a drop-out rate of 17.6%. Of the remaining 14 patients, 9 patients (64%) showed improvements in terms of pain, dysgeusia and xerostomia, while 5 patients did not notice any relevant effect. No patients reported a worsening of symptoms after using piperine.

No patients reported excessive alcohol consumption nor a history of alcohol dependence. One patient reported being a former smoker, while two patients reported current smoking of approximately 10 cigarettes per day.

At baseline, from the questionnaire on pain characteristics, it emerged that 13 out 17 patients (76.4%) reported xerostomia, all patients reported burning and widespread pain of the oral mucosa, and 4 patients (23.5%) reported dysphagia, while 12 patients (70.5%) reported dysgeusia characterized by the constant presence of a bitter or metallic taste on the tongue and palate.

### 3.2. Primary Outcomes

**Figure 1:**
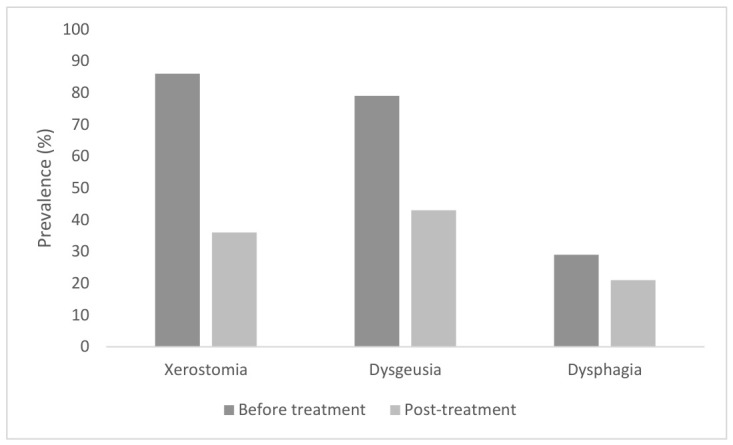
Comparison of the prevalence of BMS symptoms before and after topical piperine use for 8 weeks (expressed as %; *n* = 14 post-treatment).

Considering the 14 patients who concluded the treatment and presented at 8 weeks follow-up, overall, 9 of 14 patients (64%) reported some improvement in BMS symptoms; five patients (36%) reported that symptoms remained unchanged, while no patients reported worsening symptoms. Five patients (36%) reported xerostomia at follow-up; one patient reported symptoms in other areas of the body, while dysphagia was present in three patients. Five patients (36%) reported dysgeusia. Figure 1 describes the improvements, in particular those related to the prevalence of xerostomia and dysgeusia, after the piperine use in patients who completed the therapy.

**Figure 2:**
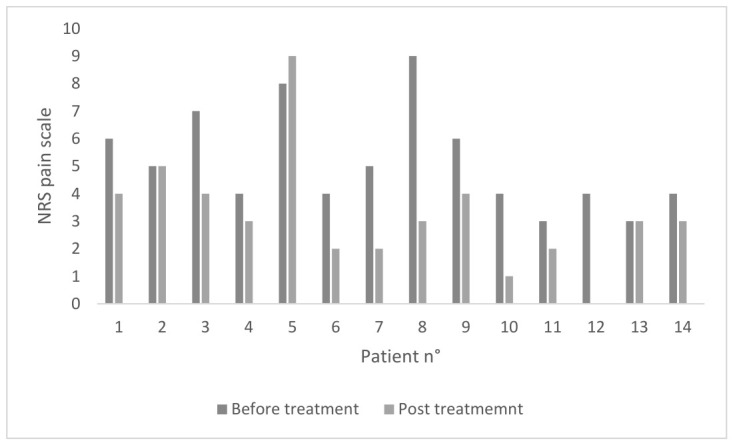
Comparison of NRS pain scale values of each patient who completed the treatment, before and after the use of topical piperine for 8 weeks (*n* = 14) (n° = number).

At NRS analysis, to evaluate the intensity of pain perceived by the patients, after 8 weeks of piperine treatment, a significant reduction was observed, with mean values of the pain scale decreasing from 5.10 ± 1.81 at baseline to 3.21 ± 2.12 post-treatment (mean difference = 1.93 ± 1.82; 95% CI 0.88 to 2.98; Shapiro–Wilk normality test of the differences, *p* = 0.182; paired samples *t*-test, *p* = 0.0016). Figure 2 describes the improvement in pain perception in the 14 patients who completed the treatment. Eight patients, in particular, achieved a clinically meaningful improvement, defined as a decrease of ≥2 points in the NRS score.

### 3.3. Secondary Outcomes

OHIP-14 mean scores significantly decreased after therapy compared with the baseline, passing from 17.6 ± 8.9 at baseline to 12.3 ± 10.4 post-treatment (mean difference = 5.21 ± 5.51; 95% CI −8.39 to −2.03). Normality of the differences was assessed using the Shapiro–Wilk test (*p* = 0.013), indicating a non-normal distribution. Therefore, the Wilcoxon signed-rank test was applied, showing a significant difference between time points (exact *p* = 0.003). Five (35.7%) patients out of 14 had no changes in the values, while 9 patients (i.e., 64.3%) reported a lower score. Table 1 shows the differences, pre- and post-treatment, among the seven OHIP-14 domains. Statistical differences were found in particular for domains related to functional limitation, physical pain, psychological discomfort and psychological disability, and social disability.

Considering the quality of sleep, the mean ESS score obtained at baseline was 3.86 ± 3.16, while the mean value at follow-up was 2.86 ± 2.96 (mean difference = 1.00 ± 1.21; 95% CI 0.30 to 1.70). A Shapiro–Wilk normality test of the differences indicated a non-normal distribution (*p* = 0.003), and the Wilcoxon signed-rank test showed a significant difference between time points (exact *p* = 0.015). No patient reported major adverse reactions or side-effects following topical piperine therapy. Three patients experienced slight irritation of the oral mucosa in the minutes following piperine application, but this did not reduce adherence to the treatment.

## 4. Discussion

In this case series, we reported the effects of topical piperine on patients with burning mouth syndrome (BMS), focusing on pain reduction, oral-health-related quality of life, and sleep disturbances. Piperine treatment significantly reduced pain intensity, as reflected by decreases in NRS scores (from 5.10 ± 1.81 at baseline to 3.21 ± 2.12 post-treatment), and improved both OHIP-14 and ESS scores, indicating enhancements in oral-health-related quality of life and daytime sleepiness. Importantly, no adverse effects were reported, and local administration was well tolerated, with only transient, mild oral discomfort immediately after rinsing.

Mechanistically, piperine acts as a TRPV1 receptor agonist, binding within the same ligand-binding pocket as capsaicin but via a distinct mode. Unlike capsaicin, which relies on canonical residues T551 and E571, piperine activation depends on T671 in the pore-forming S6 segment [27]. Activation of the channel results in prolonged opening of the receptor, which is associated with the desensitization of nerve fibers and a subsequent improvement in burning, with a mechanism similar to capsaicin, but potentially with fewer side-effects. In addition to its antinociceptive effects, piperine has demonstrated antimicrobial activity against a range of oral microorganisms, including both bacterial and fungal species [28]. Emerging evidence suggests that alterations in the oral microbiome may play a contributory role in the pathogenesis of BMS, potentially through mechanisms involving mucosal inflammation, altered sensory signaling, or interactions with peripheral nerve endings [29,30]. In this context, the antimicrobial properties of piperine may further contribute to symptom modulation not only through direct effects on nociceptive pathways, but also via indirect modulation of the oral microbial environment. Although this hypothesis remains speculative, it provides a plausible additional mechanism that warrants further investigation in future controlled studies. in our case series, the effects of topical piperine in reducing BMS symptoms, however, varied among participants, and this may be related to the complex pathophysiology of the disease, which includes peripheral and/or central causes: piperine may play a role in managing pain with peripheral pathogenesis, consistent with its local action on TRPV1-expressing nerve fibers in the oral mucosa. In our cohort, 64.3% of patients reported symptomatic improvement, and no patients experienced worsening, suggesting a favorable benefit–risk profile.

To date, clinical studies on piperine for the management of BMS and neuropathic pain are lacking. Comparing our findings with previous literature on capsaicin, topical capsaicin has demonstrated pain reduction in randomized trials on BMS, with repeated application of capsaicin gel (0.01–0.025%) or rinse (0.02%), reducing VAS scores compared with the baseline [31,32]. The use of capsaicin mucoadhesive patches (0.025 mg/cm^2^) also provided long-lasting relief from burning in BMS [9], while systemic capsaicin, though showing effects in managing BMS in a pilot study, is associated with gastric pain that limits its tolerability [33]. Overall, systematic reviews have shown only modest and short-term effects, with methodological limitations related to the studies and large heterogeneity [6]. When compared with capsaicin, piperine may be associated with greater tolerability, as confirmed by the absence of reported adverse events, minimal discomfort and good patient compliance. The improvements observed in OHIP-14 and ESS scores suggest that topical piperine may also be associated, because of the better management of burning, with a better quality of life and sleep. Limitations of this case series include the small sample size and the absence of a control group, as well as the lack of adequate long-term follow-up after treatment cessation to clarify whether the effects are lasting or only temporary.

This study was designed as an exploratory case series without a control group; therefore, it is not intended to establish efficacy but rather to generate preliminary observations on the potential role of topical piperine in the management of burning mouth syndrome (BMS). Given the well-documented susceptibility of BMS to placebo responses, the absence of a comparator arm represents an important methodological limitation that must be considered when interpreting the outcomes. The findings of this case series should thus be interpreted with caution due to the uncontrolled design, which precludes any causal inference regarding the efficacy of topical piperine. The observed improvements may, at least in part, reflect non-specific therapeutic factors rather than a true pharmacological effect. These results should therefore be considered hypothesis-generating and highlight the need for well-designed randomized controlled trials to determine the true efficacy and clinical utility of topical piperine in BMS. Additionally, no blinding was implemented, representing a further weakness that may have introduced observation bias; several potential confounding factors, including comorbidities, concomitant treatments and psychological variables, could not be controlled, and this may have further influenced the observed outcomes. Moreover, the small sample size and the absence of a priori sample size calculation, which represent further intrinsic limitations of case series, hinder the strength and generalizability of the findings. Overall, these methodological constraints limit the internal validity of the findings and preclude any causal inference.

Although our results suggest that piperine is a well-tolerated and promising topical agent for symptom relief in BMS, identifying potential dosage for topical use, future studies should focus on placebo-controlled trials, with standardized outcomes and adequate follow-up to validate these preliminary findings.

## 5. Conclusions

Burning mouth syndrome remains a highly debilitating condition with limited effective treatment options; natural compounds, including piperine extracted from *Piper nigrum*, represent therapeutic alternatives worth exploring further. In this case series, topical piperine (10 mg twice daily for 8 weeks) applied to the oral mucosa showed promise in alleviating BMS symptoms in over 50% of cases, also improving sleep and quality of life, although responses were heterogeneous across patients and there was a lack of a control group. While not all participants responded to treatment, a substantial proportion of patients showed a reduction in symptoms, supporting the potential effect of piperine in BMS management. Despite the exploratory nature of this study and the inability to establish efficacy, piperine may represent a promising therapeutic option for some patients with BMS, warranting further investigation. Studies with larger sample sizes and control groups are warranted to better define responder profiles and further explore these findings.

## Figures and Tables

**Table 1:** Description of the seven OHIP-14 domains before and after topical piperine use for 8 weeks (*n* = 14 post-treatment; Wilcoxon signed-rank test was applied for statistical analysis, considering a non-normal distribution in the Shapiro–Wilk normality test of the differences).

	Questions	Before Treatment (Mean ± SD)	Post Treatment (Mean ± SD)	*p*-Value
1. Functional limitation	Difficult pronounce words (Q1); worsened taste (Q2)	1.8 ± 1.5	1.0 ± 1.4	0.001
2. Physical pain	Pain (Q3); uncomfortable to eat (Q4)	1.4 ± 1.4	0.8 ± 1.2	0.003
3. Psychological discomfort	Self-conscious (Q5); feel tensed (Q6)	1.2 ± 1.4	0.9 ± 1.3	0.031
4. Physical disability	Diet unsatisfactory (Q7); interrupted meals (Q8)	0.3 ± 0.6	0.3 ± 0.6	1
5. Psychological disability	Difficult to relax (Q9); embarrassed (Q10)	1.4 ± 1.6	1.4 ± 1.7	0.001
6. Social disability	Irritable (Q11); difficult to do jobs (Q12)	1.2 ± 1.4	0.8 ± 1.3	0.03
7. Handicap	Life less satisfying (Q13); totally unable to function (Q14)	1.6 ± 1.8	1.3 ± 1.5	0.062
OHIP-14 mean scores	Q1–Q14	17.6 ± 8.9	12.3 ± 10.4	0.003

## Data Availability

The original contributions presented in this study are included in the article. Further inquiries can be directed to the corresponding author.

## Abbreviations

The following abbreviations are used in this manuscript:

BMS	Burning Mouth Syndrome
NRS	Numeric Rating Scale
OHIP-14	Oral Health Impact Profile
ESS	Epworth Sleepiness Scale
